# Double-carbapenemase-producing *Enterobacteriaceae*: complete genome sequencing of isolates from hospitals in Paraguay, 2021

**DOI:** 10.17843/rpmesp.2025.422.14293

**Published:** 2025-06-23

**Authors:** Nancy Melgarejo Touchet, Natalie Weiler, Sofía Busignani, Verónica Orrego, María José Duarte, Jazmin Martínez, Pamela Dunjo, Mario Martínez Mora, Aníbal Kawabata, Juan Irala, Mariel Brítez, Federico Escobar, Fátima Gonzalez, Carmen Riquelme, Beatriz Soilan

**Affiliations:** 1 Laboratorio Central de Salud Pública, Asunción, Paraguay. Laboratorio Central de Salud Pública Asunción Paraguay; 2 Hospital del Trauma “Dr. Manuel Giani”, Asunción, Paraguay. Hospital del Trauma “Dr. Manuel Giani” Asunción Paraguay; 3 ANALIZA Laboratory, Asunción, Paraguay. ANALIZA Laboratory Asunción Paraguay; 4 AMSA Sanatorium, Asunción, Paraguay. AMSA Sanatorium Asunción Paraguay; 5 Social Security Institute. Central Hospital, Asunción, Paraguay. Social Security Institute Central Hospital Asunción Paraguay; 6 Ciudad del Este Regional Hospital. Ciudad del Este, Paraguay. Ciudad del Este Regional Hospital Ciudad del Este Paraguay; 7 Hospital General de Luque. Luque, Paraguay. Hospital General de Luque Luque Paraguay; 8 National Institute of Respiratory and Environmental Diseases. Asunción, Paraguay. National Institute of Respiratory and Environmental Diseases Asunción Paraguay

**Keywords:** Klebsiella pneumoniae, Enterobacter cloacae, carbapenem resistance, dual production of carbapenemases, Whole genome sequencing, Illumina Miseq, Paraguay

## Abstract

**Objectives.:**

To describe the whole genome sequencing of double-carbapenemase-producing *Enterobacteriaceae* isolates circulating in Paraguay.

**Materials and methods.:**

We conducted genomic studies on seven *Enterobacteriaceae* isolates, previously confirmed as double-carbapenemase producers by PCR, from patients with extended hospital stays and broad-spectrum antimicrobial treatment in seven hospitals in Paraguay. Genome sequencing included Unicycler assembly and multilocus sequence typing (MLST).

**Results.:**

Of the seven Enterobacterales isolates producing dual carbapenemases, six were *Klebsiella pneumoniae* subsp. *pneumoniae* and one was *Enterobacter cloacae* subsp. *cloacae*. The co-production of *bla*_KPC-2_/*bla*_NDM-1_ and *bla*_KPC-2_/*bla*_NDM-5_ was confirmed in *K. pneumoniae*. We found co-production of *bla*_NDM-1_/*bla*_OXA-163_ in *E. cloacae*, along with other antimicrobial resistance genes of chromosomal and plasmid origin. The MLST sequence types of the *K. pneumonia*e isolates were ST11, ST15, ST133, ST273, and ST1303, and that of *E. cloacae* was ST976. Two of the six *K. pneumoniae* ST11 isolates, from two different hospitals in the capital, were genetically related and both carried *bla*_KPC-2_ and *bla*_NDM-5_.

**Conclusions.:**

We report the first genome sequencing of double-carbapenemase-producing Enterobacterales from patients with extended hospital stays in Paraguay. The analysis revealed diverse resistance profiles and clones, carriage of multiple carbapenemases, and other resistance genes of chromosomal and plasmid origin. These findings emphasize the need to strengthen hospital infection control and implement effective therapeutic interventions.

## INTRODUCTION

In recent decades, antimicrobial resistance (AMR) has become prevalent due to its worldwide increase in most microorganisms. During the COVID-19 pandemic, this situation worsened due to the excessive and inappropriate use of broad-spectrum antimicrobials, with an increase in the incidence of healthcare-associated infections reported by several low- and high-income countries ^1^. The microorganisms most affected by the acquisition of resistance to multiple drugs, including broad-spectrum drugs, are Gram-negative bacilli, particularly members of the *Enterobacteriaceae* order ^2^.

Carbapenem-resistant Enterobacteriaceae (CRE) are associated with increased morbidity and mortality. In 2017, the World Health Organization published a list of priority pathogens resistant to antimicrobials, which was updated in 2024 to include this bacterial group among the 12 most dangerous families for human health, placing them in priority 1 (critical) ^3^. *Klebsiella pneumoniae*, a recognized opportunistic pathogen, is the most important agent within the group, causing serious nosocomial infections such as sepsis, pneumonia, and urinary tract infections, among others. It plays a major role in hospital infections, increasing hospitalization time, cost, and mortality ^4^.

The main mechanism responsible for resistance is the production of carbapenemases, enzymes that destroy carbapenems, broad-spectrum β-lactam agents used to treat bacterial infections caused by multidrug-resistant Gram-negative bacilli ^5^. Currently, there are three classes of enzymes according to the Ambler classification: class A, serine carbapenemases, whose most important representative is KPC; class B, metallo-β-lactamases, which includes NDM, IMP, and VIM; and class D, serine OXA-type enzymes (OXA-48-like) ^6^.

In Paraguay, the most prevalent enzyme in this bacterial group is the NDM genotype metallo-beta-lactamase ^7^, confirmed in 2012 in isolates of *Acinetobacter pittii*^8^, which displaced KPC, confirmed in 2009 in isolates of *Enterobacter cloacae*; both are endemic in all hospitals ^9^.

Researchers from different parts of the world report the presence of more than one type of carbapenemase in a single isolate of enterobacteria. With the COVID-19 pandemic, these findings increased and were the subject of a regional alert in 2021 ^10^.

In Paraguay, in June 2021, the Central Public Health Laboratory (LCSP), a national referral laboratory, reported an increase in the number of isolates of Gram-negative bacteria resistant to broad-spectrum antimicrobials, and in September of the same year, the circulation of double-carbapenemase-producing Enterobacteriaceae in several hospitals in the country was confirmed ^11^^,^^12^.

Since these findings are of national importance, genomic studies were carried out on some double-carbapenemase-producing Enterobacteriaceae isolates in order to generate detailed knowledge about the nature of the genes involved in broad-spectrum antimicrobial resistance.

**KEY MESSAGES**

Motivation for the study. To generate knowledge about the current situation of antimicrobial resistance in Enterobacteriaceae using whole genome sequencing.

Main findings. This study presents the first genome sequencing of double-carbapenemase-producing Enterobacteriaceae from patients with extended hospital stays in Paraguay. Of the seven double-carbapenemase-producing Enterobacteriaceae isolates, six were *Klebsiella* subsp *pneumoniae*.

Implications. Our findings highlight the urgent need to strengthen measures to prevent and control healthcareassociated infections in order to prevent the spread of these highly resistant bacteria.

## MATERIALS AND METHODS

Retrospective study carried out in the Department of Bacteriology and Mycology of the LCSP, on seven double-carbapenemase-producing Enterobacteriaceae isolates from seven hospitals collaborating with the Antimicrobial Resistance Laboratory Surveillance Network. Table 1 shows the dates of isolation and the characteristics of the referral centers.

**Table 1:** Characteristics of the centers referring double-carbapenemase-producing Enterobacteriaceae isolates submitted for whole genome sequencing in Paraguay in 2021.

Strain number	Date of sample collection (d/m/y)	Region of origin	Center characteristics
1	13/03/2021	Capital	Private Hospital
2	24/07/2021	Capital	Private Hospital
3	17/08/2021	Capital	Referral Hospital
4	17/09/2021	X Health Region	Regional Hospital
5	25/08/2021	XVIII Health Region	General Hospital
6	12/07/2021	Capital	Referral Hospital
7	3/03/2021	Capital	Referral Hospital

### Confirmation of bacterial identification

Bacterial identifications were confirmed by comparing protein spectra generated by matrix-assisted laser desorption/ionization time-of-flight mass spectrometry (MALDI-TOF MS) using the BD™ Bruker MALDI Biotyper® CA System, following the manufacturer’s instructions.

### Molecular confirmation of double carbapenemase production

We used multiple endpoint polymerase chain reaction (PCR) using specific primers for the detection of the *bla*_KPC_, *bla*_NDM_, *bla*_IMP_, *bla*_VIM_, and *bla*_OXA_-48-like genes ^13^.

DNA was obtained using the bacterial lysis method by boiling a bacterial suspension of approximately 0.5 Mac Farland in 300 µL of RNA-free water for 10 minutes and then centrifuging at 10,000 rpm for 10 minutes. Gene amplification reactions were performed in a TC-PRO thermocycler (BOECO Germany) and the amplification products were analyzed by agarose gel electrophoresis in TAE buffer (PanReac AppliChem - ITW Reagents). The electrophoretic pattern images were obtained using Gel DocTM EZ Imager photo-documentation equipment (BIO-RAD) and analyzed using Image Lab 6.0 software (BIO-RAD).

### Genomic studies by massive sequencing of short molecules

Short reads were performed using the Illumina Miseq platform with the Miseq Reagent Kit V2. Libraries were prepared using the DNA library prep kit (Illumina) following the manufacturer’s instructions.

### Bioinformatic analysis

The obtained sequences were subjected to quality control using the Fastqc program and subsequently assembled using the Unicycler (SPAdes) program ^14^. The genomes were annotated using Prokka (version 3.2.1) ^15^.

Multilocus sequence typing (MLST) was performed using the Ariba software (version 2.14.4) ^16^^)^ and the PubMLST database (https://pubmlst.org). The following genes were analyzed: (*adk*, *fumC*, *gyrB*, *icd*, *mdh*, *purA*, and *recA*). We searched for resistance mechanisms in the following databases: Res_Finder (v.4.1) ^17^^)^ and Point_fonder ^18^^)^ using the software Ariba and Staramr ^19^; with threshold requirements of 95% identity and minimum coverage of 80%, in addition to the AMRfinder database, with the AMRfinder program ^20^, using the same parameters.

The search for incompatibility plasmids was performed using the Staramr program with the Plasmid_finder database (version 2021-07-12) using the parameters of 95% identity and 60% minimum coverage.

Finally, we constructed a maximum likelihood phylogenetic tree using the RAxML program ^21^ based on the pangenome study to establish the relationship between the studied isolates. The phylogenetic tree was visualized using the FigTree program (version 1.4.4) developed by Andrew Rambaut. The results were integrated with the associated metadata using the Microreact program ^22^.

### Ethical considerations

The research protocol was approved by the Research Ethics Committee of the LCSP of the MSPyBS, and received a favorable opinion from CEI-LCSP No. 187/2021.

## RESULTS

Of the seven double-carbapenemase-carrying Enterobacteriaceae isolates, six corresponded to *K. pneumoniae* subsp. *pneumoniae* (*K. pneumoniae*) and one to *Enterobacter cloacae* subsp. *cloacae* (*E. cloacae*), all from patients who had been hospitalized for more than 30 days in different healthcare centers accross the country. All *K. pneumoniae* isolates were found to carry *bla*_NDM_ + *bla*_KPC_, and the *E. cloacae* isolate carried *bla*_NDM_ + *bla*_OXA 163_. Table 2 describes the epidemiological characteristics of the studied isolates.

**Table 2:** Characteristics of double-carbapenemase-producing Enterobacteriaceae isolates subjected to whole genome sequencing in Paraguay, 2021.

Strain No.	Hospitalization center	Age in years (sex)	Length of stay in days	Disease/ diagnosis	Antimicrobial treatment	Sample (infection or colonization)	Conventional multiple PCR genotypic result	Outcome
1	Capital	61 (Woman)	150	Post-COVID-19 complications	SCF, VAN, TGC, COL, AMK, LVX, ERT, CIP, MEM, FLU.	purulent skin discharge (infection)	*K. pneumoniae bla*_NDM_ + *bla*_KPC_	Deceased
2	Capital	59 (Man)	37	SARS-CoV-2-associated pneumonia	CRO, LVX, TZP, VAN, MEM, TGC.	sputum (infection)	*K. pneumoniae bla*_NDM_ + *bla*_KPC_	Discharged
3	Capital	35 (Woman)	73	Injuries from a traffic accident.	CFZ, AMX, CIP, TZP, VAN, COL, AMK, MEM, TGC, CAZ.	abdominal discharge (infection)	*K. pneumoniae bla*_NDM_ + *bla*_KPC_	Discharged
4	Alto Paraná	46 (Man)	70	Cardiorespiratory arrest following asphyxia due to foreign body	COL, MEM, OTR.	purulent skin discharge (infection)	*K. pneumoniae bla*_NDM_ + *bla*_KPC_	Discharged
5	Central	57 (Man)	48	Rectal tumor	VAN, MEM, LIN, COL, TGC.	catheter tip (infection)	*E. cloacae bla*_NDM_ + *bla*_OXA 163_	Deceased
6	Capital	49 (Woman)	35	SARS-CoV-2-associated pneumonia	CRO, COL, AMK, MEM, FLU.	tracheal secretion (infection)	*K. pneumoniae bla*_NDM_ + *bla*_KPC_	Deceased
7	Capital	76 (Woman)	33	Chronic heart failure	CRO, MEM, IMI, TGC, COL.	rectal swab (colonization)	*K. pneumoniae bla*_NDM_ + *bla*_KPC_	Deceased

SCF: cefoperazone/sulbactam, VAN: vancomycin, TGC: tigecycline, AMK: amikacin, LVX: levofloxacin; ERT: ertapenem; CFZ: cefazolin; CAZ: ceftazidime; CIP: ciprofloxacin; MEM: meropenem; FLU: fluconazole; CRO: ceftriaxone; TZP: piperacillin/tazobactam; COL: colistin; OTR: other unregistered.

Five cases were reported in healthcare centers in the country’s capital; one in the Central Department and one in the Alto Paraná Department, located 20 and 350 km from the capital, respectively.

The results of the antimicrobial susceptibility profile study revealed the resistance of the isolates to multiple drugs, including 2 of the 7 isolates with a colistin MIC greater than 8 ug/mL. With regard to tigecycline, only 1 presented a MIC ≥8 ug/mL. Table 3 details the results of the antimicrobial susceptibility testing.

**Table 3:** Results of antimicrobial susceptibility testing of double-carbapenemase-producing Enterobacteriaceae isolates subjected to whole genome sequencing in Paraguay, 2021.

Isolate	Minimum inhibitory concentration (MIC) (ug/mL)												Inhibition halo (mm)
	CTX	CAZ	SAM	PTZ	ERT	IMI	MEM	GEN	SXT	CIP	TGC	COL*	AMK
1	≥64	≥64	≥32	≥128	4	4	8	≥16	≥16	≥4	≥8	>8	18
2	≥64	≥64	≥32	≥128	≥8	8	≥16	≥16	≥16	≥4	≤1	>8	6
3	≥64	≥64	≥32	≥128	≥8	≥16	≥16	≥16	≥16	2	≤0.5	0,5	6
4	≥64	≥64	≥32	≥128	≥8	≥16	≥16	≥16	≥16	≥4	1	0,5	17
5	≥64	≥64	≥32	≥128	≥8	≥16	≥16	≥16	≥16	2	1	≤0.25	17
6	≥64	≥64	≥32	≥128	≥8	≥16	≥16	≥16	≥16	≥4	2	0,5	6
7	≥64	≥64	≥32	≥128	≥8	≥16	≥16	≥16	≥16	1	≤0.5	≤0.25	6

Methodologies: Minimum inhibitory concentration (MIC) by automated VITEK 2C and broth microdilution (*); inhibition halos by Kirby Bauer.

CTX: cefotaxime; CAZ: ceftazidime; SAM: ampicillin/sulbactam; ERT: ertapenem; PTZ: piperacillin/tazobactam; IMI: imipenem; MEM: meropenem; GEN: gentamicin; CIP: ciprofloxacin; TGC: tigecycline; SXT: trimethoprim/sulfamethoxazole; COL: colistin; AMK: amikacin.

The whole genome sequencing results are summarized in Table 4, which shows the sequence types (ST), virulence factors, plasmids, and antimicrobial resistance genes of the isolates studied.

**Table 4:** Results of whole genome sequencing of Enterobacteriaceae isolates carrying carbapenemases in Paraguay, 2021.

	Strain 1	Strain 2	Strain 3	Strain 4	Strain 5	Strain 6	Strain 7
Strain	*K. pneumoniae*	*K. pneumoniae*	*K. pneumoniae*	*K. pneumoniae*	*E. cloacae*	*K. pneumoniae*	*K. pneumoniae*
ST	273	11	1303	15	976	11	133
Plasmids	IncFII (K) IncC IncFIB (pNDM-Mar) IncFIB(K) IncHI1B (pNDM-MAR) IncFIB(pQiI) Col(pHAD28)	IncFII(K) Col(pHAD28) Col440I IncFIB(K) Inc(pQil) IncFII	IncFIA(HI1) IncFIB(pNDM-Mar) IncX3 IncFIB(K) IncHI1B (pNDM-MAR)	IncFIB(pQil) IncFII(K) IncFIB(pNDM-Mar) IncHI1B (pNDM-MAR)	IncFII(Yp) IncFIB(pECLA) IncR	IncFII IncFIB(pQil) IncFIB(K) IncFII(K) Col(pHAD28) Col4401 IncFIB(pNDM-Mar) IncHI1B (pNDM-MAR)	FIA (pBK30683) IncC IncFIB(K) IncM1 IncR
Virulence factors RmpADC/rmpA2	N	N	N	N	N	N	N
Yersiniabactin factor	ybt 9, ICEKp3 YbST 183	ytb, ICEKp3 YbST 183-1LV	N	ytb 1, ICEKp4 YbST 28-1LV	N	ytb 9, ICEKp3 YbST 183-1LV	N
R to β-lactams	*bla*_KPC_2_	*bla*_KPC_2_	*bla*_KPC_2_	*bla*_KPC_2_	--	*bla*_KPC_2_	*bla*_KPC_2_
	*bla*_NDM_1_	*bla*_NDM_5_	*bla*_NDM_5_	*bla*_NDM_5_	*bla*_NDM_1_	*bla*_NDM_5_	*bla*_NDM_5_
	*bla*_OXA_1_ *bla*_OXA_5_	*bla*_OXA_1_ *bla*_OXA_5_	*bla*_OXA_1_ *bla*_OXA_5_	*bla*_OXA_1_ *bla*_OXA_5_	*bla*_OXA_1_ *bla*_OXA_5_ *bla*_*OXA_163*_	*bla*_OXA_1_ *bla*_OXA_5_	--
	*bla*_CTX_M_15_	*bla*_CTX_M_15_	*bla*_CTX_M_15_	*bla*_CTX_M_15_	*bla*_CTX_M_15_	*bla*_CTX_M_15_	--
	*bla*_TEM_1C_	*bla*_TEM_1B_	*bla*_TEM_1B_	--	*bla*_TEM_1B_	*bla*_TEM_1B_	*bla*_TEM_1B_
	*bla*_SHV_11_ *bla*_SHV_67_	*bla*_SHV_12_	*bla*_SHV_12_	*bla*_SHV_12_ *bla*_SHV_28_ *bla*_SHV_100_	--	*bla*_SHV_12_ *bla*_SHV_187_	*bla*_SHV_75_
	--	--	--	--	*bla*_CMH-3_	--	--
R to quinolones	qnrB_1	qnrB_1	qnrB_7	qnrB_1	--	qnrB_1	--
	--	--	--	--	--	--	qnrB_3
	--	--	--	--	--	--	qnrB_6
	oqxA	oqxA	--	OqxA	OqxA	OqxA	OqxA
	oqxB	oqxB	--	OqxB	OqxB	OqxB	OqxB
	--	--	--	--	oqxR_V113I	--	--
	qnrS_1	qnrS_1	qnrS_1	qnrS_1	qnrS_1	qnrS_1	--
	gyrA_S83I	gyrA_S83I	--	gyrA_S83I gyrA_S83F gyrA_D87A	--	gyrA_S83I	--
	parC_S80I	parC_S80I	--	parC_S80I	--	parC_S80I	--
R to Fosfomycin	fosA_5	fosA_1 fosA_6	fosA_1 fosA_6	fosA_1 fosA_6	fosA_7	fosA_1 fosA_6	fosA_1 fosA_6
R to sulfamethoxazole	sul_1	sul_1	sul_1	sul_1	--	sul_1	sul_1
	sul_2		sul_2	sul_2	sul_2	--	sul_2
	aac(6’)-Ib-cr_5	aac(6’)-Ib-cr_5	aac(6’)-Ib-cr_5	aac(6’)-Ib-cr_5	aac(6’)-Ib-cr_5	aac(6’)-Ib-cr_5	aac(6’)-Ib-cr_5
R to aminoglycosides	aph(3’’)-Ib	aph(3’)-Ia	aph(3’’)-Ib	aph(3’’)-Ib	aph(3’’)-Ib	--	aph(3’’)-Ib
	aph(6)-Id	--	aph(6)-Id	aph(6)-Id	aph(6)-Id	--	aph(6)-Id
	aac_3_II	-	aac_3_II	aac_3_II	aac_3_II	--	--
	aadA_2	aadA_2	aadA_2	aadA_2	--	aadA_2	aadA_16
	--	rmtB_1	rmtB_1	--	--	rmtB_1	rmtB_1
R to trimethoprim	dfrA_12	dfrA_12	dfrA_12	dfrA_12	--	dfrA_12	--
	dfrA_14	--	dfrA_14	--	dfrA_14	--	--
	--	--	--	--	--	--	dfrA_2 dfrA_27
R to erythromycin/ clindamycin	erm_B	erm_B	--	--	--	erm_B	--
R to erythromycin/ azithromycin	mph_A	mph_A	--	mph_A	mph_A	mph_A	--
R to chloramphenicol	catB_3	catB_3	catB_3	catB_3	catA_2 catB_3	catB_3	--
R to tetracycline	tet_A	--	--	--	--	--	tet_A tet_D
R to polymyxins	mgrB_W20R pmrB_R256G	pmrB_R256G phoQ_L96P	--	--	--	pmrB_R256G	--

ST: sequence type, R: resistance; N: negative

**Figure 1:**
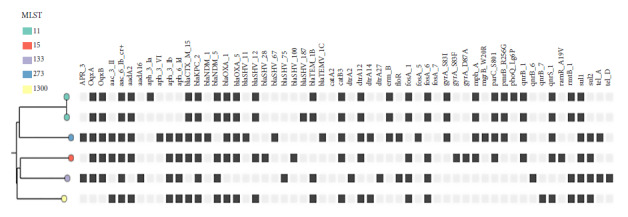
Clonal relationship between double-carbapenemase-producing *K. pneumoniae* isolates isolated in hospitals in Paraguay. Year 2021.

Only two of the six *K. pneumoniae* isolates had the same sequence type (ST11) and showed a close genetic relationship with 18 SNPs of difference, both from two hospitals in the country’s capital, where the dual production of the carbapenemases *bla*_KPC-2_ and *bla*_NDM-5_ was confirmed. One isolate from the interior of the country (ST15) was found to carry the *bla*_NDM-5_ and *bla*_KPC-2_ genes; another (ST273) carried *bla*_NDM-1_ and *bla*_KPC-2_; and the remaining two (ST133 and ST1303) carried *bla*_NDM-5_ and *bla*_KPC-2_. The only isolate of *E. cloacae* (ST976), a double-carbapenemase producer, was found to carry the *bla*_NDM-1_ + *bla*_OXA-163_ genes. The clonal relationship between the isolates is shown in Figure 1.

The extended-spectrum β-lactamases identified in the studied strains were *bla*_CTX-M-15_ and *bla*_SHV-12_; *bla*_CTX-M-15_ was found in 6 of the 7 studied strains and coexisted with SHV-12 in 4 of them.

Several mechanisms of resistance to quinolones were identified, both chromosomal and plasmid in nature. All isolates were found to carry a combination of several of these mechanisms.

We found that 100% of isolates were resistant to trimethoprim and sulfamethoxazole due to the presence of *sul* (*sul_1, sul_2*) and *dfrA* (*dfrA_2, dfrA_12, dfrA_14, dfrA_27*); and resistance to aminoglycosides due to the presence of horizontally transferred genes, evidenced in all of them by the carriage of *aac-(6´)-Ib-cr_1*, in combination with others, such as *aac_3_II, aadA*, *aph*, and *rmtB*.

None of the isolates were found to carry the *mcr* gene, however, two of them (strains 1 and 2) with colistin MIC >8 ug/mL were positive for at least two mutations (mgrB_W20R/pmrB_R256G and pmrB_R256G/phoQ_L96P), which could be the cause of colistin resistance.

The only virulence factor identified in four isolates was yersiniabactin (ybt); all were negative for virulence factors associated with hyper-muco-viscosity (RmpA/rmpA2).

## DISCUSSION

The identification of two related *K. pneumoniae* ST11 isolates, despite their origin in the capital of Paraguay, suggests possible clonal spread but limits definitive conclusions about transmission within healthcare centers. Similarly, the single isolate of *E. cloacae* restricts our ability to draw conclusions about the diversity of carbapenemases in this species. The study’s exclusive focus on double-carbapenemase producers may overlook other significant resistance mechanisms or single carbapenemase producers circulating in Paraguay.

This study presents the first whole genome sequencing analysis of double carbapenemase-producing Enterobacteriaceae isolates in Paraguay, recovered from patients with extended hospital stays in 2021. Our findings show the circulation of several Enterobacteriaceae clones carrying multiple carbapenem-hydrolyzing enzymes and several antimicrobial resistance genes, acquired through both chromosomal and plasmid mechanisms.

All isolates were from patients with extended hospital stays and high mortality rate, and all received broad-spectrum antimicrobial combination therapy. Co-production of _*blaKPC-2*_ /*bla*_NDM-1_ and *bla*_KPC-2_/*bla*_NDM-5_ was found in *K. pneumoniae*, and *bla*_NDM-1_/*bla*_OXA-163_ in *E. cloacae*. Sequence types included ST11, ST15, ST133, ST273, and ST1303 for *K. pneumoniae*, and ST976 for *E. cloacae*. Phylogenetic analysis revealed genetically unrelated species, with only two ST11 isolates from the capital exhibiting clonality, sharing plasmids, virulence factors, and resistance genes. This is likely due to the fact that this is a referral hospital for COVID-19.

The ST15 strain of *K. pneumoniae*, co-producing *bla*_KPC-2_ and *bla*_NDM-5_, was isolated from a tertiary referral hospital in the department of Alto Paraná, on the border with Brazil. This sequence type has been widely recognized as a high-risk clone due to its propensity to acquire hybrid plasmids carrying resistance and hypervirulence genes ^23^. Brazilian studies have documented the circulation of *K. pneumoniae* ST15 in several regions. For example, in 2020, Martins *et al*. reported an outbreak in São Paulo involving ST15 isolates harboring *bla*_KPC-2_, *bla*_CTX-M-15_, *bla*_SHV-28_, and other resistance determinants ^24^. Similarly, Rodríguez *et al*. described the spread of *bla*_KPC-2_-carrying ST15 in the state of Pará in 2021 ^25^. In contrast, European studies have associated ST15 with the carriage of *bla*_NDM-1_ and *bla*_OXA-48_, along with other resistance genes ^26^.

The only isolate of *E. cloacae* (ST976), a double producer of carbapenemases *bla*_NDM-1_ and *bla*_OXA-163_, was referred from a hospital in the Central Department and exhibited a wide range of antimicrobial resistance determinants. It is worth noting that this is the first report of the *bla*_OXA-163_ gene, a variant of *bla*_OXA-48_, in Enterobacterales in Paraguay. This genotype has been documented in several regional ^27^^,^^28^^)^ and global settings, associated with outbreaks ^29^. In addition, the presence of *bla*_CMH-3_, a recently described AmpC-type beta-lactamase, was confirmed. Its characteristics have not yet been fully elucidated, although there is history of association with the carbapenemase *bla*_NDM-1_^30^.

The stability of double-carbapenemase-producing isolates has been reported by several researchers. For example, Gao *et al*. demonstrated high stability in *K. pneumoniae* isolates co-producing *bla*_KPC-2_ and *bla*_NDM-1_^31^. This stability raises concerns about potential horizontal spread, highlighting the critical need to strengthen ongoing surveillance and reinforce infection prevention and control measures in healthcare settings.

In May 2021, Ahmed *et al*. reported the isolation of a hypervirulent strain of *K. pneumoniae* in an Egyptian hospital, carrying the *bla*_NDM-1_ carbapenemase gene on one plasmid and *bla*_KPC-2_ on another ^32^. Similarly, Chinese researchers documented hypervirulent isolates of *K. pneumoniae*, including ST11 strains, harboring multiple resistance genes, such as *bla*_KPC-2_^33^. ST11 is a highly epidemic clonal complex known to facilitate the horizontal transfer of multidrug-resistant Gram-negative bacteria. These findings contribute to our understanding of the ability of these microorganisms to harbor diverse plasmids, linking several determinants of resistance and virulence.

Yersiniabactin, a siderophore recognized as a virulence factor in *K. pneumoniae*, particularly in pulmonary infections ^34^, was detected in four of the six isolates. However, none of the isolates carried rpmA/rpmA2, the genes responsible for hyper-muco-viscosity in hypervirulent strains of *K. pneumoniae*^35^.

Our findings are limited by the small sample size, which restricts their generalizability to the Paraguayan population as a whole. The retrospective nature of this study, which is based on pre-existing isolates, limited access to detailed clinical and epidemiological data. The focus on isolates from patients with extended hospital stays (more than 30 days) may have introduced selection bias, which could overrepresent hospital-acquired infections and underestimate community-acquired cases. The geographic distribution of the isolates, mostly from the capital city, may not accurately reflect the prevalence and diversity of resistance mechanisms in all regions of the country. The resistance to colistin and tigecycline in a subset of isolates requires investigation with larger sample sizes to determine the extent of resistance to these last-line antibiotics.

In conclusion, our findings provide information on circulating resistance mechanisms, highlighting the usefulness of whole genome sequencing in characterizing multidrug-resistant bacteria. The data could facilitate infection control strategies and personalized therapeutic approaches, especially in high-risk hospital settings. These findings emphasize the need for surveillance and prevention and control measures for healthcare-associated infections to mitigate the spread of highly resistant pathogens.
