# Supplementary material for: Double-carbapenemase-producing Enterobacteriaceae: complete genome sequencing of isolates from hospitals in Paraguay, 2021
Source: Rev Peru Med Exp Salud Publica. 2025 Jun 23;42(2):138–46. doi: 10.17843/rpmesp.2025.422.14293 (PMC12377889; doi:10.17843/rpmesp.2025.422.14293)
Supplement: Translation

# Translation: *Enterobacteriaceae* productoras de doble carbapenemasa: secuenciación del genoma completo de aislamientos en hospitales de Paraguay, 2021

**Authors:** Nancy Melgarejo Touchet, Natalie Weiler, Sofía Busignani, Verónica Orrego, María José Duarte, Jazmin Martínez, Pamela Dunjo, Mario Martínez Mora, Aníbal Kawabata, Juan Irala, Mariel Brítez, Federico Escobar, Fátima Gonzalez, Carmen Riquelme, Beatriz Soilan

**Affiliations:** 1 Laboratorio Central de Salud Pública, Asunción, Paraguay.; 2 Hospital del Trauma “Dr. Manuel Giani”, Asunción, Paraguay.; 3 Laboratorio ANALIZA, Asunción, Paraguay.; 4 Sanatorio AMSA, Asunción, Paraguay.; 5 Instituto de Previsión Social. Hospital Central, Asunción, Paraguay.; 6 Hospital Regional de Ciudad del Este. Ciudad del Este, Paraguay.; 7 Hospital General de Luque. Luque, Paraguay.; 8 Instituto Nacional de Enfermedades Respiratorias y del Ambiente. Asunción, Paraguay.

**Keywords:** Klebsiella pneumoniae, Enterobacter cloacae, resistencia a carbapenemes, doble producción de carbapenemasas, secuenciación de genoma completo, Illumina Miseq, Paraguay

## Abstract

**Objetivos.:**

Describir la secuenciación del genoma completo de aislados de Enterobacterales dobles productores de carbapenemasas circulantes en Paraguay.

**Materiales y métodos.:**

Se realizaron estudios genómicos en siete aislamientos de Enterobacterales, previamente confirmados como dobles productores de carbapenemasas por PCR, de pacientes con estadías hospitalarias prolongadas y tratamiento antimicrobiano de amplio espectro en siete hospitales de Paraguay. La secuenciación del genoma incluyó el ensamblaje de Unicycler, la tipificación de secuencias multilocus (MLST).

**Resultados.:**

De los siete aislamientos de Enterobacterales dobles productores de carbapenemasas, seis fueron de *Klebsiella pneumoniae* subsp. *pneumoniae* y uno de *Enterobacter cloacae* subsp. *cloacae*. En *K. pneumoniae* se confirmó la coproducción de *bla*_KPC-2_ */ bla*_NDM-1_ y *bla*_KPC-2_ */ bla*_NDM-5_. En *E. cloacae* se observó la coproducción de *bla*_NDM-1_ */bla*_OXA-163_, junto con otros genes de resistencia a los antimicrobianos de origen cromosómico y plásmido. Los tipos de secuencia MLST de los aislados de *K. pneumoniae* fueron ST11, ST15, ST133, ST273 y ST1303, y de *E. cloacae* fue ST976. Dos de los seis aislados de *K. pneumoniae* ST11, procedentes de dos hospitales diferentes de la capital, estaban genéticamente relacionados, y ambos portaban *bla*_KPC-2_ y *bla*_NDM-5_.

**Conclusiones.:**

Se presenta la primera secuenciación del genoma de Enterobacterales dobles productores de carbapenemasas, de pacientes con estancias hospitalarias prolongadas en Paraguay. El análisis reveló diversos perfiles de resistencia y clones, portación de múltiples carbapenemasas y otros genes de resistencia de origen cromosómico y plasmídico. Estos hallazgos enfatizan la necesidad de fortalecer el control de infecciones hospitalarias y realizar intervenciones terapéuticas efectivas.

## INTRODUCCIÓN

En las últimas décadas, la resistencia a los antimicrobianos (RAM) tomó predominio, por su aumento evidenciado a nivel mundial en la mayoría de los microorganismos. Durante la pandemia por COVID-19 dicha situación empeoró, debido al uso excesivo e indebido de antimicrobianos de amplio espectro, observándose el incremento en la incidencia de infecciones asociadas a la atención en salud, reportados por varios países, tanto de bajos como de altos ingresos ^1^. Los microorganismos más afectados por la adquisición de resistencia a múltiples fármacos, incluso de amplio espectro, son los bacilos gramnegativos, especialmente los miembros del orden *Enterobacterales*^2^.

Las Enterobacterias resistentes a los carbapenémicos (ERC) están asociadas a mayor morbilidad y mortalidad. En el año 2017, la Organización Mundial de la Salud, publicó un listado de patógenos prioritarios resistentes a los antimicrobianos; el cual fue actualizado en el 2024; incluyendo a este grupo bacteriano entre las 12 familias más peligrosas para la salud humana, ubicándolas en la prioridad 1 (crítica) ^3^; siendo *Klebsiella pneumoniae*, reconocido oportunista, el agente más importante dentro del grupo, causante de infecciones nosocomiales graves, como sepsis, neumonías, infecciones del tracto urinario, entre otros; desempeñando un papel preponderante en las infecciones hospitalarias, aumentando el tiempo de hospitalización, costo y mortalidad ^4^.

El principal mecanismo responsable de la resistencia es por producción de carbapenemasas, enzimas que destruyen los carbapenémicos, agentes β-lactámicos de amplio espectro utilizados para el tratamiento de las infecciones bacterianas ocasionadas por bacilos gramnegativos resistentes a múltiples fármacos ^5^. En la actualidad, según la clasificación de Ambler, son conocidas tres clases de enzimas: la de clase A, serin carbapenemasas, cuya representante más importante es la KPC; la de clase B, metalo-β-lactamasas, que incluye la NDM, IMP y VIM y la de clase D, serin enzimas de tipo OXA (OXA-48-like) ^6^.

En Paraguay, la enzima prevalente en este grupo bacteriano es la de tipo metalobetalactamasa genotipo NDM ^7^, confirmada en año 2012 en aislamientos de *Acinetobacter pittii*^8^, la cual desplazó a KPC, confirmada en el 2009 en aislamientos de *Enterobacter cloacae*; ambas endémicas en todos los hospitales ^9^.

Investigadores de diferentes partes del mundo publicaron la portación de más de un tipo de carbapenemasa en un solo aislamiento de enterobacteria. Con la pandemia por COVID-19 este tipo de hallazgos se incrementó y fue motivo de una alerta regional en el año 2021 ^10^.

En Paraguay, en el mes de junio del 2021, el Laboratorio Central de Salud Pública (LCSP); laboratorio de referencia nacional; comunicó el incremento en el número de aislamientos de bacilos gramnegativos resistentes a antimicrobianos de amplio espectro, y en septiembre del mismo año, confirmó la circulación de *Enterobacterales* dobles productores de carbapenemasas en varios hospitales del país ^11^^,^^12^.

Ante estos hallazgos de relevancia nacional, se llevaron a cabo los estudios genómicos de algunos aislamientos de Enterobacterales dobles productores de carbapenemasas, a fin de generar conocimientos detallados sobre la naturaleza de los genes involucrados en la resistencia a los antimicrobianos de amplio espectro.

**MENSAJE CLAVE**

Motivación para realizar el estudio. Generar conocimientos sobre la situación actual de la resistencia antimicrobiana en Enterobacterales, utilizando secuenciación de genoma completo.

Principales hallazgos. Se presenta la primera secuenciación del genoma de Enterobacterales dobles productores de carbapenemasas, de pacientes con estancias hospitalarias prolongadas en Paraguay. De los siete aislamientos de Enterobacterales dobles productores de carbapenemasas, seis fueron de *Klebsiella* subsp pneumoniae.

Implicancias. Los hallazgos resaltan la urgencia de fortalecer las medidas de prevención y control de las infecciones asociadas a la atención de la salud, para prevenir la diseminación de estas bacterias altamente resistente.

## MATERIALES Y MÉTODOS

Estudio retrospectivo llevado a cabo en el departamento de Bacteriología y Micología del LCSP, en 7 aislamientos de Enterobacterales dobles productores de carbapenemasas, provenientes de 7 centros hospitalarios colaboradores de la Red de Vigilancia Laboratorial de la Resistencia a los Antimicrobianos. En la Tabla 1 se detallan las fechas de aislamiento y las características de los centros remitentes.

**Tabla 1:** Características de los centros remitentes de aislamientos de Enterobacterales dobles productores de carbapenemasas sometidos a secuenciación de genoma completo en Paraguay 2021.

Número de cepa	Fecha de toma de muestra	Región de procedencia	Característica del centro
1	13/03/2021	Capital	Hospital Privado
2	24/07/2021	Capital	Hospital Privado
3	17/08/2021	Capital	Hospital de Referencia
4	17/09/2021	X Región Sanitaria	Hospital Regional
5	25/08/2021	XVIII Región Sanitaria	Hospital General
6	12/07/2021	Capital	Hospital de Referencia
7	3/03/2021	Capital	Hospital de Referencia

### Confirmación de la identificación bacteriana

Las identificaciones bacterianas fueron confirmadas por comparación de espectros proteicos, generados por la técnica de espectrometría de masas de tiempo de vuelo con desorción/ionización láser asistida por matriz (MALDI-TOF MS), utilizando el Sistema BD™ Bruker MALDI Biotyper ® CA, siguiendo las instrucciones del fabricante.

### Confirmación molecular de doble producción de carbapenemasas

Se llevó a cabo por reacción en cadena de la polimerasa (PCR) múltiple de punto final utilizando cebadores específicos para la detección de los genes *bla*_*KPC,*_ *bla*_NDM_, *bla*_IMP_, *bla*_VIM_ y *bla*_OXA-48-like_^13^.

Para la obtención del ADN se utilizó el método de lisis bacteriana por ebullición durante 10 minutos de una suspensión bacteriana de aproximadamente 0,5 *Mac Farland* en 300 uL de agua libre de RNAsa y centrifugada posteriormente a 10.000 rpm por 10 minutos. Las reacciones de amplificación de los genes se realizaron en un termociclador TC-PRO (BOECO Germany) y los productos de amplificación se analizaron mediante electroforesis en gel de agarosa al 2% en tampón TAE buffer (PanReac AppliChem - ITW Reagents). Las imágenes de los patrones electroforéticos fueron obtenidas con el equipo fotodocumentador Gel Doc^TM^ EZ Imager (*BIO-RAD*) y analizadas con el programa Image Lab 6.0 (*BIO-RAD*).

### Estudios genómicos por secuenciación masiva de moléculas cortas

Las lecturas cortas se realizaron utilizando la plataforma *Illumina Miseq* con el kit Miseq Reagent Kit V2. Las librerías fueron preparadas utilizando el DNA library prep kit (*Illumina*) siguiendo las indicaciones del fabricante.

### Análisis bioinformático

Las secuencias obtenidas fueron sometidas a control de calidad con el programa Fastqc y posteriormente ensambladas con el programa Unicycler (SPAdes) ^14^. Los genomas fueron anotados con prokka (versión 3.2.1)^15^.

La tipificación por multilocus sequence typing (MLST) se realizó con el software ariba (versión 2.14.4) ^16^ utilizando la base de datos PubMLST (https://pubmlst.org). Los genes analizados fueron los siguientes: (*adk, fumC, gyrB, icd, mdh, purA, and recA*). La búsqueda de mecanismos de resistencia se realizó utilizando las siguientes bases de datos: Res_Finder (v.4.1) ^17^ y Point_fonder ^18^ mediante los softwares ariba y Staramr ^19^; con requerimientos de umbral de 95% de identidad y cobertura mínima del 80%, además de la base de datos AMRfinder, con el programa AMRfinder ^20^, utilizando los mismos parámetros.

La búsqueda de plásmidos de incompatibilidad se realizó utilizando el programa Staramr con la base de datos Plasmid_finder (versión 2021-07-12) utilizando los parámetros de 95% de identidad y 60% de cobertura mínima.

Finalmente se construyó un árbol filogenético por máxima verosimilitud con el programa RAxML ^21^ basado en el estudio del pangenoma para establecer la relación entre los aislamientos estudiados. La visualización del árbol filogenético se realizó con el programa FigTree (versión 1.4.4) desarrollado por Andrew Rambaut. La integración de los resultados junto con la metadata asociada se llevó a cabo por el programa Microreact ^22^.

### Consideraciones éticas

El protocolo de investigación fue aprobado por el Comité de Ética en Investigación del LCSP del MSPyBS, y cuenta con dictamen favorable CEI-LCSP N°187/2021.

## RESULTADOS

De los 7 aislamientos de Enterobacterales dobles portadores de carbapenemasas, 6 correspondieron a *K. pneumoniae* subsp. *pneumoniae* (*K. pneumoniae*) y 1 a *Enterobacter cloacae* subsp. *cloacae* (*E. cloacae*), todos provenientes de pacientes con estancia hospitalaria de más de 30 días en diferentes centros sanitarios del país. Todos los aislamientos de *K. pneumoniae* resultaron ser portadores de *bla*_NDM_ + *bla*_KPC_, y el de *E. cloacae* de *bla*_NDM_ + *bla*_OXA 163_. La Tabla 2 describe las características epidemiológicas de los aislamientos estudiados.

**Tabla 2:** Características de los aislamientos de Enterobacterales dobles productores de carbapenemasas sometidos a secuenciación de genoma com-pleto en Paraguay, 2021

Cepa Nro.	Centro de internación	Edad en años (sexo)	Tiempo de internación en días	Patología/ diagnóstico	Tratamiento antimicrobiano	Muestra (infección o colonización)	Resultado genotípico de PCR convencional múltiple	Desenlace
1	Capital	61 (Mujer)	150	Complicaciones post COVID-19	SCF, VAN, TGC, COL, AMK, LVX, ERT, CIP, MEM, FLU.	secreción purulenta de piel (infección)	*K. pneumoniae bla*_NDM_ + *bla*_KPC_	Fallecido
2	Capital	59 (Hombre)	37	Neumonía asociada a SARS-CoV-2	CRO, LVX, TZP, VAN, MEM, TGC.	esputo (infección)	*K. pneumoniae bla*_NDM_ + *bla*_KPC_	Alta médica
3	Capital	35 (Mujer)	73	Traumatismo por accidente de tránsito.	CFZ, AMX, CIP, TZP, VAN, COL, AMK, MEM, TGC, CAZ.	secreción abdominal (infección)	*K. pneumoniae bla*_NDM_ + *bla*_KPC_	Alta médica
4	Alto Paraná	46 (Hombre)	70	Paro cardiorrespiratorio post asfixia por cuerpo extraño	COL, MEM, OTR.	secreción purulenta de piel (infección)	*K. pneumoniae bla*_NDM_ + *bla*_KPC_	Alta médica
5	Central	57 (Hombre)	48	Tumor rectal	VAN, MEM, LIN, COL, TGC.	punta de cateter (infección)	*E. cloacae bla*_NDM_ + *bla*_OXA 163_	Fallecido
6	Capital	49 (Mujer)	35	Neumonía asociada a SARS-CoV-2	CRO, COL, AMK, MEM, FLU.	secreción traqueal (infección)	*K. pneumoniae bla*_NDM_ + *bla*_KPC_	Fallecido
7	Capital	76 (Mujer)	33	Insuficiencia cardiaca crónica	CRO, MEM, IMI, TGC, COL.	hisopado rectal (colonización)	*K. pneumoniae bla*_NDM_ + *bla*_KPC_	Fallecido

SCF: cefoperazona/sulbactam, VAN: vancomicina, TGC: tigeciclina, AMK: amicacina, LVX: levofloxacina; ERT: ertapenem; CFZ: cefazolina; CAZ: ceftazidima; CIP: ciprofloxacina; MEM: meropenem; FLU: fluconazol; CRO: ceftriaxona; TZP: piperacilina/tazobactam; COL: colistina; OTR: otros no registrado.

Cinco aislamientos provinieron de centros asistenciales de la capital del país; 1 del Dpto. Central y 1 del departamento Alto Paraná, distantes a 20 y 350 km de la capital del país respectivamente.

Los resultados del estudio del perfil de susceptibilidad a los antimicrobianos estudiados, reveló la resistencia de los aislamientos a múltiples drogas, incluso 2 de los 7 aislamientos estudiados con CIM de colistina mayor a 8 ug/mL. En lo que respecta a la tigeciclina, sólo 1 presentó CIM ≥8 ug/mL. La Tabla 3 detalla los resultados de la prueba de susceptibilidad a los antimicrobianos.

**Tabla 3:** Resultados de las pruebas de susceptibilidad a los antimicrobianos de los aislamientos de Enterobacterales dobles productores de carbapenemasas sometidos a secuenciación de genoma completo en Paraguay, 2021.

Aislamiento	Concentración Inhibitoria Mínima (CIM) (ug/mL)												Halo de inhibición (mm)
	CTX	CAZ	SAM	PTZ	ERT	IMI	MEM	GEN	SXT	CIP	TGC	COL*	AMK
1	≥64	≥64	≥32	≥128	4	4	8	≥16	≥16	≥4	≥8	>8	18
2	≥64	≥64	≥32	≥128	≥8	8	≥16	≥16	≥16	≥4	≤1	>8	6
3	≥64	≥64	≥32	≥128	≥8	≥16	≥16	≥16	≥16	2	≤0,5	0,5	6
4	≥64	≥64	≥32	≥128	≥8	≥16	≥16	≥16	≥16	≥4	1	0,5	17
5	≥64	≥64	≥32	≥128	≥8	≥16	≥16	≥16	≥16	2	1	≤0,25	17
6	≥64	≥64	≥32	≥128	≥8	≥16	≥16	≥16	≥16	≥4	2	0,5	6
7	≥64	≥64	≥32	≥128	≥8	≥16	≥16	≥16	≥16	1	≤0,5	≤0,25	6

Metodologías: Concentración Inhibitoria Mínima (CIM) por automatizado VITEK 2C y microdilución en caldo (*); halos de inhibición por Kirby Bauer.

CTX: cefotaxima; CAZ: ceftazidima; SAM: ampicilina/sulbactam; ERT: ertapenem; PTZ: piperacilina/tazobactam; IMI: imipenem; MEM: meropenem; GEN: gentamicina; CIP: ciprofloxacina; TGC: tigeciclina; SXT: trimetoprim/sulfametoxazol; COL: colistina; AMK: amicacina.

Los resultados de secuenciación de genoma completo están resumidos en la Tabla 4, en donde se pueden observar los secuenciotipos (ST), factores de virulencia, plásmidos y los genes de resistencia a los antimicrobianos de los aislamientos estudiados.

**Tabla 4:** Resultados de la secuenciación del genoma completo de aislamientos de Enterobacterales dobles portadores de carbapenemasas en Paraguay 2021.

	Cepa 1	Cepa 2	Cepa 3	Cepa 4	Cepa 5	Cepa 6	Cepa 7
Cepa	*K. pneumoniae*	*K. pneumoniae*	*K. pneumoniae*	*K. pneumoniae*	*E. cloacae*	*K. pneumoniae*	*K. pneumoniae*
ST	273	11	1303	15	976	11	133
Plásmidos	IncFII (K) IncC IncFIB (pNDM-Mar) IncFIB(K) IncHI1B (pNDM-MAR) IncFIB(pQiI) Col(pHAD28)	IncFII(K) Col(pHAD28) Col440I IncFIB(K) Inc(pQil) IncFII	IncFIA(HI1) IncFIB(pNDM-Mar) IncX3 IncFIB(K) IncHI1B (pNDM-MAR)	IncFIB(pQil) IncFII(K) IncFIB(pNDM-Mar) IncHI1B (pNDM-MAR)	IncFII(Yp) IncFIB(pECLA) IncR	IncFII IncFIB(pQil) IncFIB(K) IncFII(K) Col(pHAD28) Col4401 IncFIB(pNDM-Mar) IncHI1B (pNDM-MAR)	FIA (pBK30683) IncC IncFIB(K) IncM1 IncR
Factores de virulencia RmpADC/rmpA2	N	N	N	N	N	N	N
Factor Yersiniabactin	ybt 9, ICEKp3 YbST 183	ytb, ICEKp3 YbST 183-1LV	N	ytb 1, ICEKp4 YbST 28-1LV	N	ytb 9, ICEKp3 YbST 183-1LV	N
R a β-lactámicos	*bla*_KPC_2_	*bla*_KPC_2_	*bla*_KPC_2_	*bla*_KPC_2_	--	*bla*_KPC_2_	*bla*_KPC_2_
	*bla*_NDM_1_	*bla*_NDM_5_	*bla*_NDM_5_	*bla*_NDM_5_	*bla*_NDM_1_	*bla*_NDM_5_	*bla*_NDM_5_
	*bla*_OXA_1_ *bla*_OXA_5_	*bla*_OXA_1_ *bla*_OXA_5_	*bla*_OXA_1_ *bla*_OXA_5_	*bla*_OXA_1_ *bla*_OXA_5_	*bla*_OXA_1_ *bla*_OXA_5_ *bla*_*OXA_163*_	*bla*_OXA_1_ *bla*_OXA_5_	--
	*bla*_CTX_M_15_	*bla*_CTX_M_15_	*bla*_CTX_M_15_	*bla*_CTX_M_15_	*bla*_CTX_M_15_	*bla*_CTX_M_15_	--
	*bla*_TEM_1C_	*bla*_TEM_1B_	*bla*_TEM_1B_	--	*bla*_TEM_1B_	*bla*_TEM_1B_	*bla*_TEM_1B_
	*bla*_SHV_11_ *bla*_SHV_67_	*bla*_SHV_12_	*bla*_SHV_12_	*bla*_SHV_12_ *bla*_SHV_28_ *bla*_SHV_100_	--	*bla*_SHV_12_ *bla*_SHV_187_	*bla*_SHV_75_
	--	--	--	--	*bla*_CMH-3_	--	--
R a quinolonas	qnrB_1	qnrB_1	qnrB_7	qnrB_1	--	qnrB_1	--
	--	--	--	--	--	--	qnrB_3
	--	--	--	--	--	--	qnrB_6
	oqxA	oqxA	--	OqxA	OqxA	OqxA	OqxA
	oqxB	oqxB	--	OqxB	OqxB	OqxB	OqxB
	--	--	--	--	oqxR_V113I	--	--
	qnrS_1	qnrS_1	qnrS_1	qnrS_1	qnrS_1	qnrS_1	--
	gyrA_S83I	gyrA_S83I	--	gyrA_S83I gyrA_S83F gyrA_D87A	--	gyrA_S83I	--
	parC_S80I	parC_S80I	--	parC_S80I	--	parC_S80I	--
R a fosfomicina	fosA_5	fosA_1 fosA_6	fosA_1 fosA_6	fosA_1 fosA_6	fosA_7	fosA_1 fosA_6	fosA_1 fosA_6
R a sulfametoxazol	sul_1	sul_1	sul_1	sul_1	--	sul_1	sul_1
	sul_2		sul_2	sul_2	sul_2	--	sul_2
	aac(6’)-Ib-cr_5	aac(6’)-Ib-cr_5	aac(6’)-Ib-cr_5	aac(6’)-Ib-cr_5	aac(6’)-Ib-cr_5	aac(6’)-Ib-cr_5	aac(6’)-Ib-cr_5
R a aminoglucosidos	aph(3’’)-Ib	aph(3’)-Ia	aph(3’’)-Ib	aph(3’’)-Ib	aph(3’’)-Ib	--	aph(3’’)-Ib
	aph(6)-Id	--	aph(6)-Id	aph(6)-Id	aph(6)-Id	--	aph(6)-Id
	aac_3_II	-	aac_3_II	aac_3_II	aac_3_II	--	--
	aadA_2	aadA_2	aadA_2	aadA_2	--	aadA_2	aadA_16
	--	rmtB_1	rmtB_1	--	--	rmtB_1	rmtB_1
R a trimetoprim	dfrA_12	dfrA_12	dfrA_12	dfrA_12	--	dfrA_12	--
	dfrA_14	--	dfrA_14	--	dfrA_14	--	--
	--	--	--	--	--	--	dfrA_2 dfrA_27
R a eritromicina/ clindamicina	erm_B	erm_B	--	--	--	erm_B	--
R a eritromicina/ azitromicina	mph_A	mph_A	--	mph_A	mph_A	mph_A	--
R a cloranfenicol	catB_3	catB_3	catB_3	catB_3	catA_2 catB_3	catB_3	--
R a tetraciclina	tet_A	--	--	--	--	--	tet_A tet_D
R a polimiximas	mgrB_W20R pmrB_R256G	pmrB_R256G phoQ_L96P	--	--	--	pmrB_R256G	--

ST: secuenciotipo, R: resistencia; N: negativo.

Solo 2 de los 6 aislamientos de *K*. *pneumoniae* presentaron el mismo secuenciotipo (ST11), y mostraron una estrecha relación genética con 18 SNP de diferencia, ambos provenientes de 2 centros hospitalarios de la capital del país, en los que se confirmaron la doble producción de las carbapenemasas *bla*_KPC-2_ y *bla*_NDM-5_. Un aislamiento del interior del país (ST15), resultó ser portador de los genes *bla*_NDM-5_ y *bla*_KPC-2_; otro (ST273), con portación de *bla*_NDM-1_ y *bla*_KPC-2_; y los 2 restantes (ST133 y ST1303) de *bla*_NDM-5_ y *bla*_KPC-2_. El único aislamiento de *E. cloacae* (ST976), doble productor de carbapenemasas, resultó ser portador de los genes *bla*_NDM-1_ + *bla*_OXA-163_. La relación clonal entre los aislamientos se visualiza en la Figura 1.

**Figura 1:** Relación clonal entre aislamientos de K. pneumoniae dobles productores de carbapenemasas aislados en hospitales de Paraguay. Año 202.

Las β-lactamasas de espectro extendido identificadas en las cepas estudiadas fueron *bla*_CTX-M-15_ y *bla*_SHV-12_; encontrándose *bla*_CTX-M-15_ en 6 de las 7 estudiadas y coexistiendo con SHV-12 en 4 de ellas.

Fueron identificados varios mecanismos de resistencia a las quinolonas, tanto de naturaleza cromosómica como plasmídica. Todos los aislamientos resultaron ser portadores de la combinación de varios de ellos.

El 100% de los aislamientos fueron resistentes a trimetoprim y sulfametoxazol por la portación de *sul (sul_1, sul_2)* y *dfrA (dfrA_2, dfrA_12, dfrA_14, dfrA_27*); y la resistencia a los aminoglucosidos por la presencia de genes transferidos horizontalmente, evidenciándose en todos ellos la portación de *aac-(6´)-Ib-cr_1*, en combinación con otros, tales como *aac_3_II, aadA, aph y rmtB.*

Ninguno de los aislados resultó ser portador del gen *mcr*, sin embargo 2 de ellos (cepa 1 y 2) con CIM de colistina >8 ug/mL fueron positivos para al menos 2 mutaciones (mgrB_W20R/pmrB_R256G y pmrB_R256G/phoQ_L96P), lo cual podría ser la causa de la resistencia a la colistina.

El único factor de virulencia identificado en 4 aislamientos fue yersiniabactin (ybt); siendo todos negativos para los factores de virulencia asociados a la hipermucoviscosidad (RmpA/rmpA2).

## DISCUSIÓN

La identificación de dos aislados relacionados de *K. pneumoniae* ST11, a pesar de su origen en la capital de Paraguay, sugiere una posible propagación clonal, pero limita las conclusiones definitivas sobre la transmisión dentro de los centros de salud. De manera similar, el aislado único de *E. cloacae* restringe nuestra capacidad para sacar conclusiones sobre la diversidad de carbapenemasas en esta especie. El enfoque exclusivo del estudio en los productores de dobles carbapenemasas puede pasar por alto otros mecanismos de resistencia significativos o productores de carbapenemasas simples que circulan en Paraguay.

Este estudio presenta el primer análisis de secuenciación del genoma completo de aislados de Enterobacterales dobles productores de carbapenemasas en Paraguay, recuperados de pacientes con estadías hospitalarias prolongadas en 2021. Los hallazgos muestran la circulación de diversos clones de Enterobacterales portadores de múltiples enzimas hidrolizadoras de carbapenémicos y varios genes de resistencia a los antimicrobianos, adquiridos a través de mecanismos tanto cromosómicos como plasmídicos.

Todos los aislamientos pertenecían a pacientes con hospitalizaciones prolongadas, con una alta tasa de mortalidad, y todos recibieron terapia combinada antimicrobiana de amplio espectro. Se observó la coproducción de *bla*_KPC-2_ / *bla*_NDM-1_ y *bla*_KPC-2_ / *bla*_NDM-5_ en *K. pneumoniae*, y *bla*_NDM-1_/ *bla*_OXA-163_ en *E. cloacae*. Los tipos de secuencia incluyeron ST11, ST15, ST133, ST273 y ST1303 para *K. pneumoniae*, y ST976 para *E. cloacae*. El análisis filogenético reveló especies genéticamente no relacionadas, con solo dos aislados de ST11 de la capital que exhiben clonalidad, compartiendo plásmidos, factores de virulencia y genes de resistencia. Es probable que esto se deba a que se trata de un hospital de referencia para COVID-19.

La cepa ST15 de *K. pneumoniae*, coproductora de *bla*_KPC-2_ y *bla*_NDM-5_, fue aislada de un hospital de nivel terciario de referencia en el departamento de Alto Paraná, en la frontera con Brasil. Este tipo de secuencia ha sido ampliamente reconocido como un clon de alto riesgo debido a su propensión a adquirir plásmidos híbridos portadores de genes de resistencia e hipervirulencia ^23^. En particular, estudios brasileños han documentado la circulación de *K. pneumoniae* ST15 en varias regiones. Por ejemplo, en 2020, Martins *et al*. informaron de un brote en São Paulo en el que participaron aislados de ST15 que albergaban *bla*_KPC-2_, *bla*_CTX-M-15_, *bla*_SHV-28_ y otros determinantes de resistencia ^24^. Del mismo modo, Rodríguez *et al*. describieron la diseminación de ST15 portadora de *bla*_KPC-2_ en el estado de Pará en 2021 ^25^. Por el contrario, estudios europeos han asociado ST15 con el transporte de *bla*_NDM-1_ y *bla*_OXA-48_, junto con otros genes de resistencia ^26^.

El único aislado de *E. cloacae* (ST976), doble productor de carbapenemasas *bla*_NDM-1_ y *bla*_OXA-163_, se remitió de un hospital del Departamento Central y exhibió una amplia gama de determinantes de resistencia a los antimicrobianos. Cabe destacar que esta es la primera detección reportada del gen *bla*_OXA-163_, una variante de *bla*_OXA-48_, en Enterobacterales en Paraguay. Este genotipo se ha documentado en varios entornos regionales ^27^^,^^28^ y mundiales, asociados a brotes ^29^. Además, se confirmó la presencia de *bla*_CMH-3_, una betalactamasa de tipo AmpC recientemente descripta, cuyas características no fueron completamente descifradas aun, aunque hay antecedente de asociación con la carbapenemasa *bla*_NDM-1_^30^.

La estabilidad de los aislados dobles productores de carbapenemasas ha sido reportada por varios investigadores. Por ejemplo, Gao *et al*. demostraron una alta estabilidad en aislados de *K. pneumoniae* coproductores de *bla*_KPC-2_ y *bla*_NDM-1_^31^. Esta estabilidad suscita preocupación en cuanto a la posible diseminación horizontal, lo que pone de relieve la necesidad crítica de reforzar la vigilancia continua y reforzar las medidas de prevención y control de infecciones asociadas a la atención de la salud.

En mayo de 2021, Ahmed *et al*. informaron del aislamiento de una cepa hipervirulenta de *K. pneumoniae* en un hospital egipcio, portadora del gen *bla*_NDM-1_ carbapenemasa en un plásmido y *bla*_KPC-2_ en otro ^32^. De manera similar, investigadores chinos documentaron aislados hipervirulentos de *K. pneumoniae*, incluidas las cepas ST11, que albergan múltiples genes de resistencia, como *bla*_KPC-2_^33^. ST11 es un complejo clonal altamente epidémico conocido por facilitar la transferencia horizontal de bacterias Gram negativas multirresistentes. Estos hallazgos contribuyen a nuestra comprensión de la capacidad de estos microorganismos para albergar diversos plásmidos, vinculando varios determinantes de resistencia y virulencia.

En cuatro de los seis aislamientos examinados se detectó yersiniabactina, un sideróforo reconocido como factor de virulencia en *K. pneumoniae*, particularmente en infecciones pulmonares ^34^. Sin embargo, ninguno de los aislados portaba rpmA/rpmA2, los genes responsables de la hipermucoviscosidad en cepas hipervirulentas de *K. pneumoniae*^35^.

Los hallazgos de este estudio están limitados por el pequeño tamaño de muestra, lo que restringe su generalización a la población paraguaya en general. La naturale­za retrospectiva de este estudio, que se basa en aislamientos preexistentes, tuvo un acceso limitado a datos clínicos y epi­demiológicos detallados. El enfoque en los aislamientos de pacientes con estadías hospitalarias prolongadas (más de 30 días) puede introducir un sesgo de selección, lo que podría representar en exceso las infecciones adquiridas en el hospital y subestimar los casos adquiridos en la comunidad. La distribución geográfica de los aislados, con una concentración en la ciudad capital, puede no reflejar con precisión la prevalencia y diversidad de los mecanismos de resistencia en todas las regiones del país. La resistencia observada a la colistina y la tigeciclina en un subconjunto de aislados requiere investigación con tamaños de muestra más grandes para determinar el alcance de la resistencia a estos antibióticos de última línea.

En conclusión, los hallazgos del estudio proporcionan información sobre los mecanismos de resistencia circulantes, destacando la utilidad de la secuenciación del genoma completo en la caracterización de bacterias resistentes a múltiples fármacos. Los datos podrían facilitar a las estrategias de control de infecciones y enfoques terapéuticos personalizados, especialmente en entornos hospitalarios de alto riesgo. Estos hallazgos enfatizan la necesidad de medidas de vigilancia y de prevención y control de infecciones asociadas a la atención de la salud, para mitigar la propagación de patógenos altamente resistentes.
